# Salinity tolerances of two Australian freshwater turtles, *Chelodina expansa* and *Emydura macquarii* (Testudinata: Chelidae)

**DOI:** 10.1093/conphys/cow042

**Published:** 2016-10-15

**Authors:** Deborah S. Bower, David M. Scheltinga, Simon Clulow, John Clulow, Craig E. Franklin, Arthur Georges

**Affiliations:** 1 Institute for Applied Ecology, University of Canberra, ACT 2601, Australia; 2 Water Quality and Aquatic Ecosystem Health Branch, Department of Environment and Resource Management, GPO Box 2454, QLD 4001, Australia; 3 University of Newcastle, Callaghan, NSW 2308, Australia; 4 School of Biological Sciences, The University of Queensland, St Lucia, QLD 4072, Australia

**Keywords:** chloride, salinization, salt, sodium, tortoise, urea

## Abstract

Freshwater biota experience physiological challenges in regions affected by salinization, but often the effects on particular species are poorly understood. Freshwater turtles are of particular concern as they appear to have limited ability to cope with environmental conditions that are hyperosmotic to their body fluids. Here, we determined the physiological responses of two Australian freshwater chelid turtles, *Emydura macquarii* and *Chelodina expansa*, exposed to freshwater (0‰) and brackish water (15‰, representing a hyperosmotic environment). Brackish water is common in the Murray–Darling River Basin within the natural range of these species in Australia during periods of drought, yet it is unknown how well these species tolerate saline conditions. We hypothesized that these turtles would be unable to maintain homeostasis in the 15‰ water treatment and would suffer osmotic loss of water, increased ionic concentrations and a decrease in body mass. Results revealed that these turtles had elevated plasma concentrations of sodium, chloride, urea and uric acid in the plasma. Plasma ionic concentrations increased proportionally more in *E. macquarii* than in *C. expansa*. Individuals of both species reduced feeding in 15‰ water, indicating that behaviour may provide an additional means for freshwater turtles to limit ion/solute influx when in hyperosmotic environments. This osmoregulatory behaviour may allow for persistence of turtles in regions affected by salinization; however, growth rates and body condition may be affected in the long term. Although we demonstrate that these turtles have mechanisms to survive temporarily in saline waters, it is likely that sustained salinization of waterways will exceed their short- to medium-term capacity to survive increased salt levels, making salinization a potentially key threatening process for these freshwater reptiles.

## Abstract (precis)

Two species of Australian freshwater turtle were submerged in either water of 0‰ or 15‰ over 50 days. Turtles in 15‰ water reduced feeding and had raised plasma ionic concentrations of sodium, chloride, urea and uric acid to decrease dehydration and enable survival.

## Introduction

Freshwater organisms maintain and regulate body fluids hyperosmotic to the environment via osmoregulation, counteracting the efflux of ions and influx of water (Davenport and Macedo, 1990; Gilles and Delpire, 1997). Most freshwater vertebrate species are stenohaline; hence, they can be severely challenged by increasing environmental salinities (Hart *et al*., 1991). Some species, however, which have recently invaded freshwater, or those that evolved in conditions of fluctuating salinities, are more tolerant of saline conditions (Williams, 1999; McCormick and Bradshaw, 2006). This increase in tolerance to higher salinities influences basic ecological parameters, such as distribution and demography (e.g. growth rate; Dunson, 1986; Brischoux *et al*., 2012). In recent decades, human-induced impacts on freshwater environments have included increasing salinization of waterways as a consequence of irrigation, altered run-off patterns, sea water intrusion, tree clearance for agriculture causing increased aridity and rising saline groundwater levels (Williams, 2001; George *et al*., 2012). However, for many species there is a poor understanding of how they will respond to such increases in salinity that are substantially higher than those baseline levels to which they have been naturally exposed (Hart *et al*., 1991). Therefore, gaining an understanding of how species tolerate salt is important to predict the impacts of increasing salinity, which is a globally occurring threatening process (Williams, 1999).

Salinization is a complex problem. For example, the Murray–Darling Basin, Australia's largest drainage basin, has experienced severe increases in salinity since European settlement, with salt measurements ranging up to 22‰ (Nielsen *et al*., 2003). This has resulted in considerable alteration to the dynamics of the aquatic chemistry of the system as a result of rising saline groundwater and reduced frequency of high-flow events (Hart *et al*., 2003). Freshwater turtles are one taxa that can be very sensitive to salt (Dunson, 1981). However, one study found that wild populations of three species of Australian freshwater turtles inhabiting a brackish lake showed only a mild change in osmolytes in response to brackish water, and this may have been behaviourally regulated, for instance by drinking freshwater, or physiologically regulated via salt excretion (Bower *et al*., 2012a). Knowledge of species’ tolerance to salinity and mechanisms of persistence can assist with predicting the impacts of salinization.

Many marine and brackish reptiles (crocodiles, turtles and snakes) possess specialized adaptations (in gills, in the tongue and near eyes) to concentrate and excrete excess salts that have accumulated in body tissues. Marine and estuarine turtles have salt glands in their eyes (Babonis and Brischoux, 2012), whereas freshwater turtles generally cannot tolerate hypersaline conditions (Bentley *et al*., 1967). Many freshwater turtles lose body mass and eventually die when immersed in brackish water (Dunson and Seidel, 1986). Nevertheless, some euryhaline estuarine turtle species, such as diamondback terrapins (*Malaclemys terrapin*), can tolerate periods in hypersaline environments by physiological mechanisms, such as increasing their plasma osmotic pressure relative to that of the external environment in order to reduce water loss. Although this is achieved in part by plasma sodium and chloride increasing moderately when the animals are placed in saline water, there is an important osmotic contribution from organic osmolytes, such as urea, which increases in concentration as much as 5-fold (Gilles-Baillen, 1970). Thus, diamondback terrapins and other euryhaline turtles (species usually associated with estuarine environments) minimize water loss by maintaining a higher osmotic gradient in their blood through increased concentrations of urea or other nitrogenous compounds, such as uric acid, urates and free amino acids (Withers, 1998; Lee *et al*., 2006), when they are in saline water that exceeds the capacity of the salt gland to excrete excess ions (Gilles-Baillen, 1970).

Freshwater turtles exploiting estuarine habitats may use a combination of compensatory and evasive and behavioural mechanisms to reduce exposure to high salinity (Hart and Lee, 2006; Harden *et al*., 2015), and some species also possess clear adaptive physiological and homeostatic mechanisms to tolerate elevated saline through reducing uptake of salt and loss of water (Dunson and Mazzotti, 1989). In this study, we assessed the tolerance and physiological responses of two species of Australian freshwater turtles, *Emydura macquarii* and *Chelodina expansa*, both of which occur extensively throughout the Murray–Darling Basin in lengths of the river system far removed from the lower estuary of the Basin, to exposure to brackish water that was hyperosmotic to their body fluids. We hypothesized that these turtles would be unable to maintain homeostasis in the 15‰ water treatment and that this would be reflected through reduced feeding, leading to loss of body mass in the 15‰ water and corresponding increases in the concentration of plasma osmolytes (sodium, chloride, urea, uric acid and potassium) over time.

## Materials and methods

### Study species

*Chelodina expansa* and *E. macquarii* occupy the inland rivers of southeastern Australia's Murray–Darling Basin. Coastal populations occur in southeast Queensland (Bower *et al*., 2013), and offshore populations occur in the dune lakes of Fraser, Moreton and Stradbroke islands off the southeast coast of Queensland. *Emydura macquarii* is more broadly distributed, including the Nepean–Hawkesbury drainage in the south, the Normanby drainage in the North, and the Cooper, Diamantina, Paroo and Bulloo drainages of central Australia. Both species are highly vagile; male *C. expansa* move readily up to 33 km and females occupy a smaller home range (Bower *et al*., 2012b), and movements of *E. macquarii* can be equally far (Katie Howard, unpublished data).

### Experimental design

We collected 24 *C. expansa* and 19 *E. macquarii* from Wentworth, New South Wales (NSW), Australia, at the confluence of the Murray and Darling Rivers between 20 and 25 March 2007. The required sample size was estimated from variation in the blood chemistry of other studies on turtles (Bower *et al*., 2012a, 2013). Turtles were transported to the University of Canberra, where they were individually housed inside a building in 46 litre plastic bins (597 mm × 362 mm × 266 mm) in closed tanks at 20°C. The light cycle was 12 h–12 h light to dark. Water was changed every 2 or 3 days for the duration that they were held. The experiment was completed under the approval of University of Canberra Animal ethics CEAE07-08, and NSW National Parks and Wildlife Service licence S12504.

Straight-line carapace length of turtles was measured using callipers and allocated to a water type treatment (0 or 15‰) in alternating size measurements to minimize confounding results from size differences; equal ratios of female to male were in each treatment group (Dunson and Heatwole, 1986). *Emydura macquarii* in the 0‰ treatment had a mean ± CI (95% confidence interval) carapace length of 28.4 ± 3.4 cm (*n* = 10) and in the 15‰ treatment 26.4 ± 2.7 cm (*n* = 9). *Chelodina expansa* measured a mean ± SD of 31.1 ± 7.45 cm in 0‰ (*n* = 12) and 30.5 ± 7.0 cm in the 15‰ treatment (*n* = 12).

On 27 May 2007, turtles were placed into either a freshwater or brackish treatment. The freshwater treatment turtles were kept in tap water with a salinity of 0‰ for the duration of the experiment. Animals in the brackish treatment were acclimated by placement into water of 5, 7, 10 and 13‰ progressively every 2 days, followed by 15‰ until for 50 days, and finally returned to fresh tap water for 7 days. The experimental period was chosen to reflect what we thought would be the upper tolerance to immersion in brackish water based on the limited tolerance of freshwater turtles to saline water (Dunson and Mazzotti, 1989; Lee, *et al*., 2006). Brackish water was obtained by mixing sea water with tap water in appropriate proportions, and the resultant salinity was verified with a multiparameter water analyser (Yeokal Electronics Pty Ltd, Brookvale, NSW, Australia). All sea water was obtained from the boat ramp at South Durras, NSW, USA (35°39.453′S, 150°17.801′E). Five pieces of raw beef (mean ± SEM 8 ± 0.56 g) were offered to each individual weekly before the experiment began and on days 4, 16, 39, 50 and 54, and the number of pieces remaining the following day were recorded and removed to determine food consumption.

Prior to blood extraction, turtles were weighed (**±**0.1 g) using an Optek digital balance (Optek Technology Inc., Blairstown, NJ, USA). We obtained 0.5 ml of blood from the jugular vein of each turtle using 23 gauge (*E. macquarii*) and 25 gauge needles (*C. expansa*) attached to disposable 1 ml syringes. Blood was extracted on days 0, 12, 36, 32 and 50 and after a week in 0‰ on day 57. Coagulation was prevented by first irrigating the syringe with lithium heparin, and evacuating all but a residue so as not to dilute the blood sample appreciably. Blood was kept on ice in a plastic tube for up to 2 h, spun at 2324 g for 5 min, before plasma was extracted, transferred to sealed tubes and frozen at −80°C until laboratory testing. Plasma was assayed by a commercial pathology laboratory (Clinical Pathology Laboratory at the School of Veterinary Science, University of Queensland). An Olympus AU400 autoanalyser (Olympus, Hamburg, Germany) was used to quantify sodium, chloride, potassium, uric acid and urea.

### Data analysis

To compare the effect of immersion in different salinities, we analysed response variables (food consumption, body mass, sodium, chloride, urea, uric acid and potassium) separately using a mixed-effects general linear model with individual turtle (ID) nested within fixed effects of species (*C. expansa* or *E. macquarii*), water type (15 or 0‰) and time (day 0, 12, 36 or 50), and carapace length as a covariate because there is evidence that smaller turtles have higher concentrations of water and potassium and lower concentrations of sodium (Dunson and Heatwole, 1986). To determine whether turtles recovered over the final week in 0‰, we reran the models including only values from days 0 and 57. The change in body mass for individual animals was calculated and expressed as the percentage gain or loss in mass during each time interval in comparison to the time zero values. Analyses were completed in SAS version 8.2 (SAS Institute, Cary, NC) in accordance with the procedures outlined by Sokal and Rohlf (1981). Means are presented with standard error bars as calculated in R 3.1.2 (R Core Team, 2014) with the package psych (Revelle, 2015). Statistical significance was assumed when *P* < 0.05.

## Results

**Figure 1:**
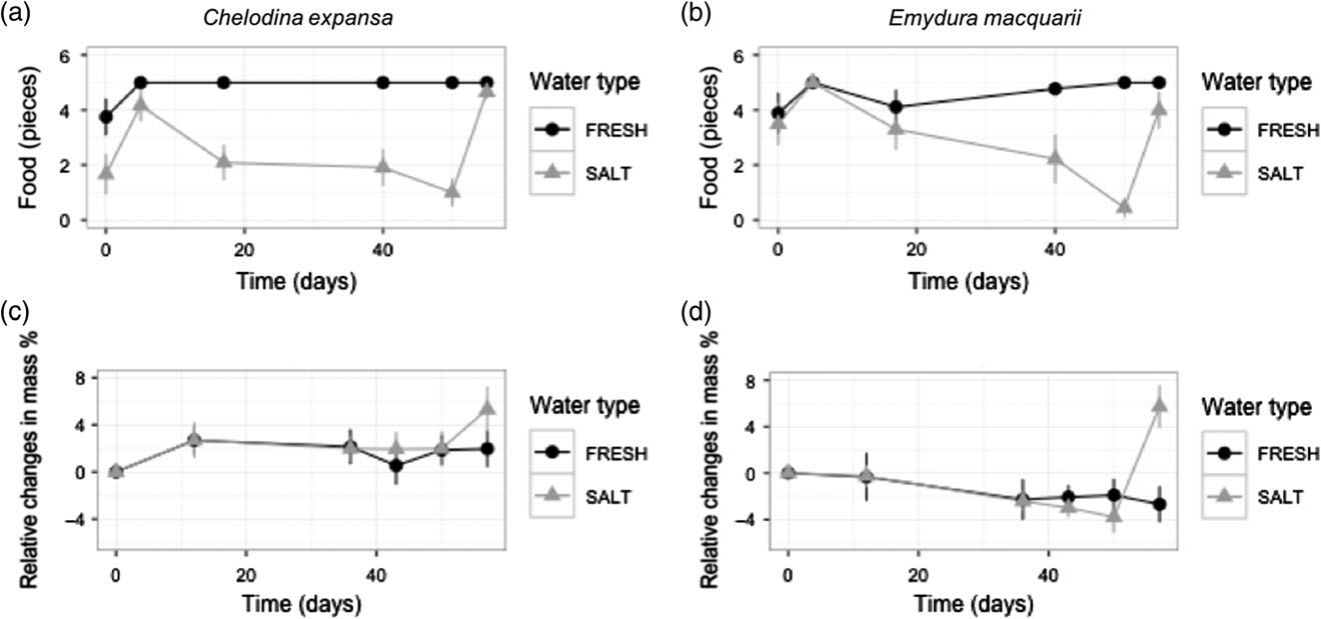
Changes in food consumption and body mass of 24 *Chelodina expansa* and 19 *Emydura macquarii* during 50 days in 0‰ water (filled circles) and 15‰ (grey triangles) treatments, followed by 7 days in 0‰ water. Values are expressed as a percentage of values at time zero.

Both species reduced feeding in 15‰ but not 0‰ (water type × time: *F*_4,125_ = 9.31, *P* < 0.0001). By 50 days, *E. macquarii* had reduced food consumption to 2.5 ± 0.2 pieces in 15‰ water treatment, whereas most food pieces were consumed by turtles in 0‰ (4.7 ± 0.1 pieces; Fig. 1a and b). *Chelodina expansa* placed in 15‰ water ate less on 0 day, consumed similar prey to 0‰ animals at 4 days and then consumed less food than 0‰ individuals at 39 and 50 days. Once returned to 0‰ for 4 days, both species increased feeding. *Emydura macquarii* in 15‰ had similar feeding intake to 0 days, but feeding remained significantly lower than turtles that had been exposed to 0‰ (water type: *F*_1,37_ = 4.53, *P* < 0.05), whereas *C. expansa* in 15‰ ate similar quantities to those in 0‰. Feeding patterns did not differ statistically between species (water type × species: *F*_1,36_ = 2.81, *P* = 0.10), and individuals with smaller carapace lengths tended to continue feeding, whereas larger individuals reduced feeding in the 15‰ water (size: *F*_1,37_ = 5.30, *P* < 0.05).

*Chelodina expansa* and *E. macquarii* survived 50 days of immersion in 15‰ water treatment. The change in body mass over the duration of the experiment did not differ significantly between turtles in 0 and 15‰ water for either species (time × water type × species: *F*_3,66.9_ = 0.48, *P* = 0.69). *Chelodina expansa* maintained an average of 3.1 ± 0.2 kg and *E. macquarii* an average of 2.2 ± 0.2 kg during 50 days of exposure to water type treatments. However, after turtles were returned to 0‰ for 7 days, individuals from both species (water type × species: *F*_1,33_ = 1.89, *P* = 0.18) increased significantly from their original mass (water type: *F*_1,33_ = 10.71, *P* < 0.01) by 5.5 ± 1.8% (Fig. 1c and d).

**Figure 2:**
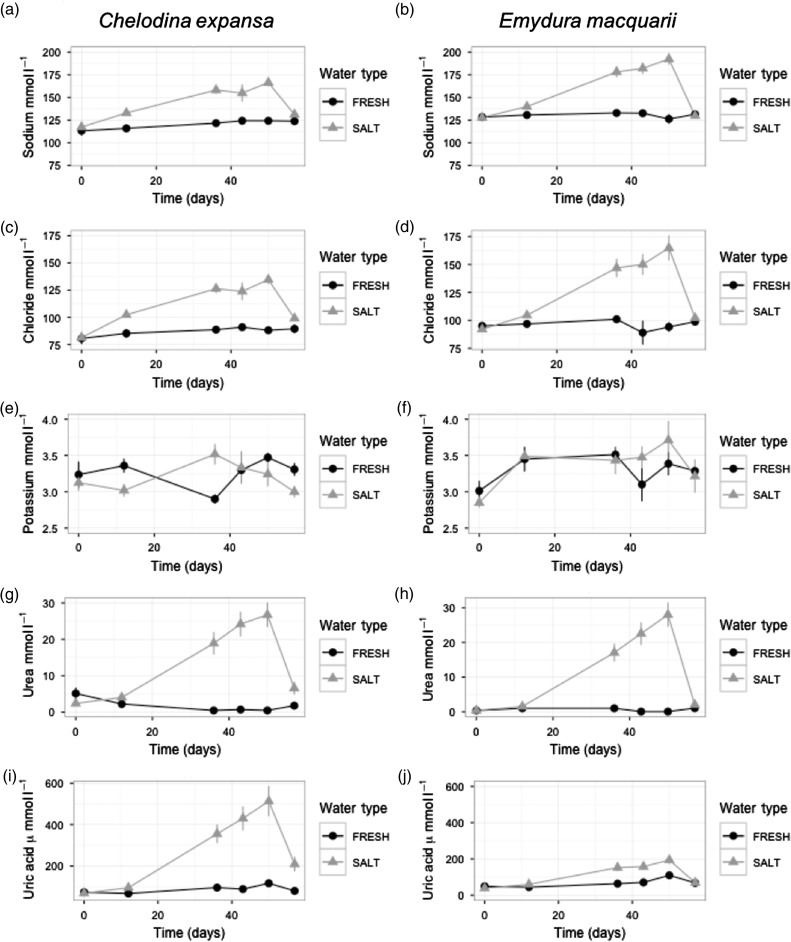
Sodium (**a** and **b**), chloride (**c** and **d**), urea (**e** and **f**) and uric acid (**g** and **h**) during 50 days in 0‰ water (filled circles) and 15‰ water treatments (grey triangles), followed by 7 days in freshwater for 24 *C. expansa* and 19 *E. macquarii*.

Sodium increased continually in turtles in 15‰ water treatment for the duration of the experiment. After 50 days in 15‰ water, the sodium concentration had increased from 117.3 ± 2.2 to 166.3 ± 4.0 mmol l^−1^ in *C. expansa.* This was more pronounced in *E. macquarii*, in which the sodium concentration increased from 127.8 ± 1.2 to 192.4 ± 6.0 mmol l^−1^ (Fig. 2a and b; water type × time × species: *F*_4,51.6_ = 5.10, *P* < 0.01). After turtles had returned to 0‰ for 7 days, sodium concentration did not differ from baseline values or among treatment types (water type × time: *F*_1,28.4_ = 0.38, *P* = 0.54).

Chloride concentration followed a similar pattern, and after 50 days of immersion in 15‰ water treatment, it increased from 81.3 ± 2.3 to 134.6 ± 3.8 mmol l^−1^ in *C. expansa*. This trend was greater in *E. macquarii*, in which the chloride concentration increased from 92.1 ± 1.7 to 164.8 ± 11.0 mmol l^−1^ (Fig. 2c and d; water type × time × species: *F*_*4*,50.6_ = 5.00, *P* < 0.01). After 7 days in 0‰, the chloride concentration of both turtle species remained significantly higher in the turtles that had previously been in 15‰ water, compared with baseline values and individuals in 0‰ (water type × time: *F*_1,33.7_ = 9.42, *P* < 0.01).

Potassium concentrations were variable and differed between species, in different water types and over time (water type × time × species: *F*_4,58.3_ = 7.23, *P* < 0.0001); these trends did not correspond to salinity differences but instead were a product of high variability attributable to individual differences (Fig. 2e and f). *Emydura macquarii* had an average potassium concentration of 3.34 ± 0.05 mmol l^−1^, and *C. expansa* was similar, at 3.25 ± 0.04 mmol l^−1^.

The largest relative changes in plasma osmolytes occurred in the concentration of urea in turtles immersed for 50 days in 15‰ water treatment (Fig. 2g and h). The urea concentration increased over time in the 15‰ water from 1.48 ± 0.33 mmol l^−1^ to a peak at 50 days of 27.22 ± 2.38 mmol l^−1^ (water type × time: *F*_4,41.1_ = 48.08, *P* < 0.0001), representing a >10-fold increase. This did not differ between species (species × time: *F*_4,37.8_ = 0.83, *P* = 0.52). The mean urea concentration of *E. macquarii* returned to baseline values after 7 days in 0‰, whereas that of *C. expansa* in 15‰ remained slightly but significantly higher than those in 0‰ (water type × time × species: *F*_1,30.8_ = 4.29, *P* < 0.05).

There was also a substantial increase in uric acid concentration (Fig. 2i and j) during 50 days of immersion in 15‰ water treatment. Uric acid increased from 38.7 ± 2.6 to 193.8 ± 21.7 µmol l^−1^ in *C. expansa*, and this trend was greater in *E. macquarii*, in which the uric acid concentration increased from a mean concentration of 68.9 ± 9.6 to 513.0 ± 71.6 µmol l^−1^ after 50 days in 15‰ water (water type × time × species: *F*_4,43.3_ = 9.53, *P* < 0.0001). The uric acid concentration of *E. macquarii* returned to baseline values after 7 days in 0‰, but the uric acid concentration of *C. expansa* exposed to 15‰ water remained significantly higher than baseline values and individuals in 0‰ (water type × time × species: *F*_1,12_ = 10.34, *P* < 0.01).

## Discussion

The results of our study demonstrated that *C. expansa* and *E. macquarii* have adaptive behavioural and physiological mechanisms reported previously only from euryhaline estuarine turtles living with periods of elevated, hypertonic environmental salinity (Dunson, 1986), despite the species studied here being widely distributed through the largest freshwater catchment in Australia. This suggests that there has been a long history of evolutionary adaptation to the fluctuating salinities of the Murray–Darling system in these species (driven by extended dry periods during droughts that cause cessation of river and stream flow and the formation of elevated salinity in pools) or that such adaptive mechanisms may be more common in freshwater species than previously expected. It is unclear from our study, and beyond its scope to determine, whether the pre-adaption to periods of hypersalinity for up to 50 days indicates that these species will retain their fitness in conditions of ongoing increases in baseline salinity associated with the longer-term impacts of European land use and climate change. Nevertheless, our study indicated a high adaptive fitness of the species to acute salinity events, from which physiological recovery appears to be rapid. Those adaptations evident in the species that we investigated include a behavioural reduction in food intake, which would reduce the ingestion of salt, which is rapidly reversed on return to freshwater. Although we did not measure osmolarity directly, the increase in plasma electrolytes (especially sodium and chloride) and nitrogenous osmolytes (urea and uric acid) would be expected to reduce the loss of water through the integument by a reduction in the transdermal osmotic gradient (Withers, 1998). The behavioural reduction in food intake may be associated with a reduced metabolic rate, because digestive activity is reduced, and is probably associated with a reduction, if not cessation, in excretion (a water-conserving mechanism), because there would be fewer metabolic substrates and waste products of metabolism and digestion.

Turtles did not lose body mass during immersion in the 15‰ treatment, indicating that the increased blood osmotic pressure was sufficient to offset osmotic water loss through the integument (Bentley *et al*., 1967). The elevated osmolarity was attributable in both species to concentrations of sodium, chloride, urea and uric acid that increased after immersion in 15‰ water. This mechanism, involving elevated plasma electrolytes and/or elevated nitrogenous osmolytes, has been identified widely in invertebrates altering osmoregulation to reduce water loss as an adaptation to variable environmental salinity (Gilles and Delpire, 1997). The reduced food consumption in the turtles exposed to the 15‰ treatment in this study is also a behavioural adaptation used in other species to reduce salt intake (Davenport and Ward, 1993). The reduction of salt intake is an important mechanism to limit dehydration that would inevitably occur in a hypersaline environment, which would occur in conditions requiring the elimination of excess sodium and chloride. The fact that these two mechanisms of generation and tolerance of elevated osmolarity and reduction in salt intake occurred together in both these species, with no evidence of ill effect, indicate a high level of tolerance to elevated saline conditions.

An important finding of our study was the maintenance of body mass during the 50 day period when feeding was greatly reduced, and the ~5% increase in body mass during the following 7 days of rehydration in 0‰ water. It is likely that the increase in mass that occurred in the turtles after removal from 15‰ water and return to 0‰ water was related to a volume expansion (plasma and extracellular fluid compartments) following rehydration, because at the same time as body mass increased, the plasma concentrations of electrolytes, urea and uric acid returned to levels similar to 0‰ (Thorson, 1968). This combination of apparent volume expansion and confirmed reduction in electrolytes and other osmolytes might indicate that there is some restriction in the rate of resumption of excretion, which is compensated for by volume expansion through the movement of newly ingested water into the available plasma and extracellular compartments (Robinson and Dunson, 1976). Potentially, such a mechanism could reduce the metabolic and physiological demands on renal function while it is upregulating in response to the altered homeostatic and hydration status of the animals. Further monitoring of turtles beyond the 7 day rehydration interval in this study may be required to investigate this phenomenon.

Nevertheless, despite the efficiency of reducing salt intake through the reduction in feeding, there may be an ecological fitness cost to the species if periods of hypersalinity persist. Although the present study showed that *C. expansa* and *E. macquarii* survived durations of several weeks in saline water, plasma osmolytes, urea and uric acid rose continuously at each sampling occasion, and whether they would do so with further prolonged duration requires further investigation. There are physiological costs associated with high osmolyte levels, e.g. metabolic demands increase in saline media, probably owing to protein degradation and urea synthesis, which could plausibly reduce fitness (Lee *et al*., 2006). At the cellular level, hypernatraemia would cause cell shrinkage if a threshold of excessive sodium uptake is exceeded (Hoffmann *et al*., 2009), and this can lead to changes in enzyme function. In addition, survival in the 15‰ treatment appeared to be at the cost of the core function of feeding. Therefore, the persistence of freshwater turtles in saline environments may be limited if they cannot access freshwater, and this finding raises the need to understand the effect of extended or more frequent periods of exposure to higher salinity. Other environment factors can have effects on blood chemistry in vertebrates, and the capacity of turtles to tolerate salt may change as a result of temperature, dormancy and acclimation (Muir *et al*., 2008; Harden, *et al*., 2015). The fitness impact of limited periods of elevated salinity (which in this study had no obvious adverse effects) may be different from higher-frequency or sustained intervals of elevated saline conditions. A loss of body condition under such regimes would be an indicator of the limitations of the adaptive fitness of the physiological mechanisms that have evolved in *C. expansa* and *E. macquarii*.

The substantial increase in urea was an effective physiological response in *C. expansa* and *E. macquarii* to increase osmotic pressure through elevated plasma concentrations of organic compounds. This is documented in a wide suite of fauna, including frogs, crocodilians and elasmobranches (Smith, 1936; Gordon *et al*., 1961; Laurén, 1985; Liggins and Grigg, 1985; Withers, 1998). In *Pelodiscus sinensis*, the rate of urea synthesis increases 1.4-fold during a 6 days of immersion in brackish water, with rapid achievment of a plasma osmolarity that can reduce dehydration (Lee *et al*., 2006). It is likely that *C. expansa* and *E. macquarii* also regulated the synthesis and excretion of urea in saline conditions. The increase in uric acid in the plasma of *C. expansa* would also have added to an elevated osmolality in that species (as opposed to *E. macquarii*, in which the increase was small), although given the low solubility of uric acid, the contribution to total osmotic pressure may have been very low. Potentially, the elevated uric acid concentration in *C. expansa* might be more indicative of a uricotelic strategy for excreting or limiting accumulation of nitrogen compounds in normal physiological conditions, and could even be a byproduct of partial renal shutdown in a species that would normally remove uric acid rapidly via the kidneys to the bladder. In contrast to other ions, potassium concentration did not change, probably because potassium is excreted effectively by the reptilian kidney and tightly controlled to maintain health (Dessauer, 1970); but see Stephens and Hauben (1989).

*Emydura macquarii* and *C. expansa* displayed similar responses in their increase in urea, although there was a comparatively greater increase in sodium, chloride and uric acid in *E. macquarii*. Differences between the species in this respect may reflect the thinner shell of *E. macquarii*, because the carapace morphology of turtles influences their resistance to ionic stress (Dunson and Heatwole, 1986). *Chelodina longicollis*, a closely related but more terrestrial species, may have an even higher tolerance to salinity than *C. expansa* and *E. macquarii* (Scheltinga, 1991; Smith, 1993; Bower *et al*., 2012a) and may also potentially be more efficient at coping with salt, owing to the possible presence of a salt-excreting orbital gland, which may assist it to occupy a niche in saline habitats (Chessman, 1984).

In conclusion, our study suggests that these two species of Australian freshwater turtles have physiological mechanisms to enable them to exist in transiently saline waters, which may be a result of their long history of co-evolution within a highly variable, arid landscape with fluctuating salinity levels in wetlands (Gell *et al*., 2007). The high variability of chemical conditions in Australian river systems has probably selected for salinity tolerance in these two aquatic reptiles, a phenomenon seen in some fish species, which also have unusually high tolerances to salinity, compared with those in other continents (Williams and Williams, 1991). Nevertheless, other factors, such as invasive species including the parasitic tubeworms in the lower Murray River, may also prove to impose a greater constraint on persistence across their current distribution than physiological limits alone (Bower *et al*., 2012a). Although these turtles have mechanisms to survive in transiently saline waters, it needs to be determined whether sustained salinization of waterways (such as that caused by anthropogenic influences) will exceed the capacity of turtles to survive increased salt concentrations, making salinization a potentially increasing threat to the livelihood/survivorship persistence of these aquatic vertebrates over their present distributions. The question remains whether future adaptation of these species will require metabolic and physiological adaptations that allow them to persist for longer periods with lower energy demands in a metabolically and physiologically suppressed state without compromising fundamental homeostatic limits.
